# Application of chitosan in fruit preservation: A review

**DOI:** 10.1016/j.fochx.2024.101589

**Published:** 2024-06-22

**Authors:** Jingjing Wang, Yuning Yuan, Yu Liu, Xiang Li, Shengjun Wu

**Affiliations:** Jiangsu Key Laboratory of Marine Bioresources and Environment/Jiangsu Key Laboratory of Marine Biotechnology, Jiangsu Ocean University, Haizhou 222005, China; Co-Innovation Center of Jiangsu Marine Bio-industry Technology, Haizhou 222005, China

**Keywords:** Chitosan, Fruit preservation, Review

## Abstract

Fruit preservation after harvest is one of the key issues in current agriculture, rural areas, and for farmers. Using chitosan to keep fruits fresh, which can reduce the harm caused by chemical preservative residue to human health. It also helps avoid the disadvantages of the high cost of physical preservation and the challenges associated with difficult operation. This review focuses on the application progress of chitosan in fruit preservation. Studies have shown that chitosan inhibits the growth of bacteria and fungi, and delays fruit aging and decay. Furthermore, it can regulate the respiration and physiological metabolism of fruit, helping to maintain its quality and nutritional value. The preservation mechanism of chitosan includes its antibacterial properties, film-forming properties, and its effects on the physiological processes of fruit. However, in practical applications, issues such as determining the optimal concentration and treatment of chitosan still require further research and optimization.

## Highlights

• Chitosan alone can effectively preserve varying fruits.
• Chitosan combination other substances can effectively preserve varying fruits.
• Modified chitosan can effectively preserve varying fruits.

## 1 Introduction

Fruits are susceptible to decay and spoilage during storage (Pace & Cefola, 2021). The reasons for its deterioration are very diverse. Such as fruit surface damage, excessively high humidity or temperature, and other factors can lead to the invasion of microorganisms (Mahanti et al., 2022; Wu, Tang, & Fan, 2024). Due to the excessively dry or poorly ventilated storage environment, certain fruits may lose water, dry out, and shrivel during storage. The epidermis of the fruit may brown due to oxidation, affecting its appearance and taste. This is usually caused by enzyme and free radical reactions in the fruit (Li & Yu, 2001; Wu, Zhang, & Fan, 2024). Inadequate low temperatures can result in cold or freezing damage to fruits, which is evident as black skin spots and soft flesh. Undesirable odors may develop during storage due to microbial metabolites or chemical changes. Fruit rot during the postharvest stage severely hinders its marketing supply chain and shelf life. Each year, approximately one-third of the fruit is wasted during the agricultural and post-harvest stages before reaching the consumer (Zhou et al., 2022). Therefore, there is a great need to develop a green and cost-effective method to extend the shelf life of perishable foods (Zhou et al., 2023).

As a novel type of preservative, the preservative has been embraced by manufacturers because of its affordable price and simple operation. With people's increasing requirements for fruit quality and safety, the use of synthetic preservatives in fruit and vegetable preservation and its potential harm have attracted more and more attention (Muthuvelu et al., 2023). Therefore, the development of new and efficient natural preservative has become a hot topic in fruit and vegetable preservation research. Compared with other traditional preservatives, chitosan has shown good performance in antioxidant, antibacterial, and other aspects. It can be combined with natural antioxidants and bacteriostasis agents to form a composite film. Its excellent anticorrosion performance fully meets the market needs. This paper introduces the corrosion mechanism, corrosion technology, and influencing factors of chitosan. The content of this article is summarized in Table 1.

**Table 1:** Summary of the contents of this article.

Chapters	Subheading	Reference
Structure of chitosan and its preparation method	Source and structure of chitosan	Fig. 1
	Preparation method of chitosan	Fig. 2
The physiological activities of chitosan	Film-forming properties	Fig. 3
	Antimicrobial properties	
	Antioxidant properties	
	Moisturizing	
	Biodegradability	
Application of chitosan in fruit preservation	Chitosan serves as a preservative alone	Triunfo et al. (2023)
		Li, Huang, et al. (2023) and Li, Sun, et al. (2023)

		Lin et al. (2020)
		Jiang et al. (2020)
		Xin et al. (2020)
	Chitosan and polysaccharide compound preservation	Zhou et al. (2024)


		Zhao et al. (2023)
		Sun et al. (2023)

		Ruan et al. (2022)
		Liu et al. (2022)
		Yuan et al. (2023)
	Chitosan and protein compound preservation	
		Qi et al. (2023)
		Zhang et al. (2019)
	Chitosan and essential oil compound preservation	
		Phuong et al. (2024)

		Pan et al. (2022)
		Yuan et al. (2023)
		Yang et al. (2023)
		Wang, Wang, et al. (2022) and Wang, Zhang, et al. (2022)
	Chitosan-nanomaterials composite preservation	


		Wang et al. (2023)

		Liu et al. (2021)
		Sami et al. (2021)
		Eldib et al. (2020)
		Xing et al. (2020)
		Qiao et al. (2019)
	Application of modified chitosan in fruit preservation	

		Jiang et al. (2024)
		Zidan et al. (2023)
		Sun et al. (2023)
		Ruan et al. (2022)
		Zhou et al. (2022)
		Pan et al. (2022)
		Fu et al. (2021)
Factors affecting the preservation effect of chitosan	Deacetylation degree	–
	pH	–
	Molecular mass and mass concentration	–

## 2 Structure of chitosan and its preparation method

### 2.1 Source and structure of chitosan

Chitosan is derived from the *N*-deacetylation of chitin. Chitin is the second most abundant polysaccharide on earth after cellulose (Liao, Hou, & Huang, 2022; Yao, Zhang, & Fan, 2023), consisting of three reactive functional groups: the amino group at the C-2 position, and the primary and secondary hydroxyl groups at C-3 and C-6, respectively. The chitin is mainly found in marine invertebrates and insects as a major component of the exoskeleton (Kumari & Kishor, 2020). Also present in certain fungi as a component of the cell wall.

**Fig. 1:**
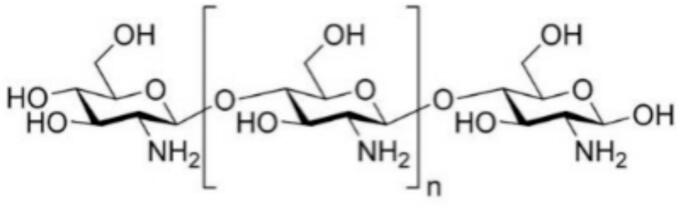
The chemical structure of chitosan.

Marine invertebrates such as crabs, shrimp, lobsters, and oysters serve as valuable sources of protein-rich marine food annually (Hajji et al., 2021). However, the shells and other inedible parts of these crustaceans constitute about half of the body mass and are usually discarded as waste (Kumar, Ye, Dobretsov, & Dutta, 2019). These wastes become an important source of chitin. Chitosan (CS) is a polysaccharide composed of *N*-acetyl-D-glucosamine and double glucosamine units (Urs, Moerschbacher, & Cord-Landwehr, 2023). It is primarily derived from the partial deacetylation of chitin, leading to the production of an *N*-acetylglucosamine and D-glucosamine copolymer. Chitosan is a soluble form of chitin and has been utilized in various industrial applications, such as food preservation and packaging (Teixeira-Costa & Andrade, 2021). The structure of the glycan is shown in “Fig. 1”.

### 2.2 Preparation method of chitosan

**Fig. 2:**
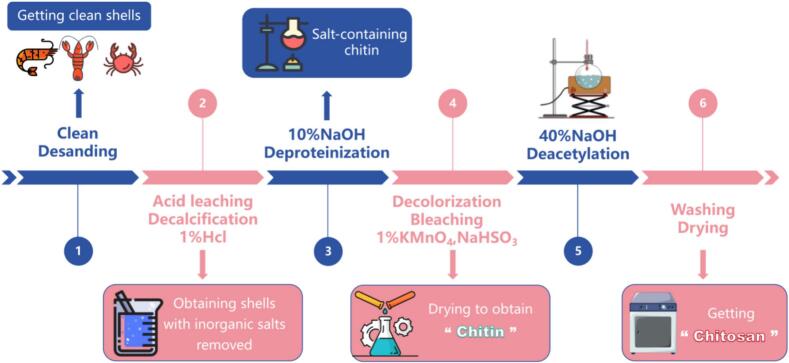
Preparation process of chitosan.

At present, there are three main methods for producing chitosan: chemical, physical, and microbial methods (Divya & Jisha, 2018). It is usually prepared by a chemical method, which involves deproteinization, desalination, and decolorization to extract chitin first, and then prepare chitosan through deacetylation and degradation processes. The traditional chemical preparation process is shown in “Fig. 2”.

## 3 The physiological activities of chitosan

**Fig. 3:**
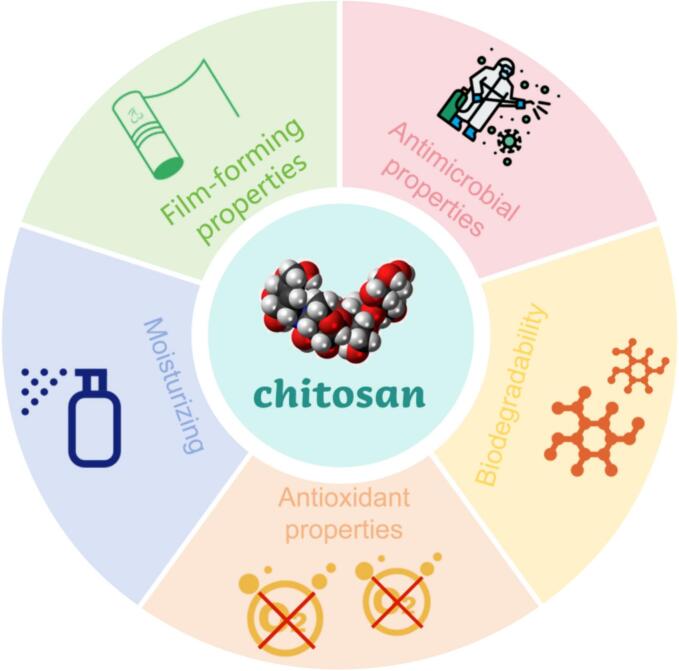
The physiological activities of chitosan.

Chitosan is non-toxic and tasteless. It is a macromolecular straight-chain polymer that can be extracted from silkworm pupa (Zhu et al., 2018), eggshell, and shells of marine organisms (Hossain & Uddin, 2020). It has good biocompatibility, wide sources, is inexpensive and easy to obtain, and environmental friendly. Chitosan itself has the advantages of antibacterial properties, film formation, and biological compatibility (Lee et al., 2023). These properties can effectively prevent fruit decay during storage, maintain its quality, and have made it a commonly used material in the study of perishable food ingredients and food plastic film. The physiological activities of chitosan are shown in “Fig. 3”.

### 3.1 Film-forming properties

The film formation of chitosan refers to the property that it can form thin films of specific thickness and strength under particular conditions. Chitosan is a natural polymer polysaccharide extracted from the shells of crustaceans such as shrimp and crab (Kou, Peters, & Mucalo, 2021). It has good film-forming properties, which are related to its molecular structure and chemical properties. Chitosan molecules contain a significant number of hydroxyl and amino groups whose functional groups can create hydrogen bonds with water molecules, allowing chitosan to form a colloidal solution in an aqueous environment. When the water in the solution gradually evaporates, the hydrogen bonding interactions between chitosan molecules are enhanced, leading to the formation of a film with specific strength and elasticity. The film-forming properties of chitosan are affected by several factors (Riseh, Vatankhah, Hassanisaadi, & Kennedy, 2023). For example, the solution concentration, pH value, temperature, solvent, etc. In general, higher solution concentrations and lower pH values favor the film formation of chitosan. In addition, incorporating crosslinkers or additives can enhance the performance of chitosan films by improving strength, transparency, water resistance, and other properties. The film-forming properties of chitosan give it a wide range of applications in various fields. The coating containing chitosan is applied to the surface of the fruit to create a transparent film. This film helps prevent fruit water evaporation, reducing weight loss and wrinkles on the fruit. At the same time, the chitosan film can also facilitate the entry of oxygen into the fruit, slowing down its respiration and metabolism, thereby extending the shelf life of the fruit. Chitosan has been widely used in the field of medicine and cosmetics. Overall, the film-forming property of chitosan is one of its significant characteristics, which expands the potential for its application in various fields (Egorov et al., 2023; Lewandowska & Szulc, 2022).

### 3.2 Antimicrobial properties

The antimicrobial effect of chitosan was first reported in 1979. It was found to have broad-spectrum antimicrobial properties that can inhibit the growth and reproduction of harmful microorganisms (Mumtaz et al., 2023), thereby prolonging the shelf-life of food products. Chitosan forms a film that blocks the exchange of internal and external substances, preventing nutrients from reaching the interior of the bacterial hyphae, which ultimately results in bacterial depletion and death. The positively charged amino groups in the chitosan molecule form inhibitory bonds, and the negatively charged cell walls of microorganisms are attracted to the positively charged amino groups, leading to rupture of the cell wall and thus cell death. Chitosan can selectively bind to various metal ions, inhibiting microbial growth and reproduction along with toxin production. The antimicrobial properties of chitosan are also affected by molecular weight and pH. In general, chitosan with a higher molecular weight and lower degree of deacetylation exhibits stronger antimicrobial properties. Under acidic conditions, chitosan is more likely to interact with the surface of bacterial cells, thereby enhancing its antimicrobial effect (Mondal, Dhar, Banerjee, Hasnain, & Nayak, 2022).

### 3.3 Antioxidant properties

Chitosan possesses antioxidant properties, which are mainly attributed to the reactive amino and hydroxyl groups in its molecular chains. It enables the substance to react with free radicals, thereby reducing their number, mitigating oxidative damage to food caused by free radicals, and delaying food deterioration (Li, Mi, Tan, & Guo, 2020). In addition, it inhibits the activity of some oxidative enzymes, such as superoxide dismutase and catalase, further slowing down the oxidation reaction. This antioxidant effect is not only helpful for fruit preservation but also important for maintaining human health, which may help prevent chronic diseases such as heart disease, cancer, and aging (Qiu, Jiang, Yan, Jiao, & Wang, 2020). It can also inhibit lipid peroxidation and protect the integrity and stability of cell membranes. The antioxidant effect of chitosan is also influenced by various factors, including its structure, molecular weight, and concentration. Different experimental conditions and systems may yield varying results. Low molecular weight chitosan exhibits stronger antioxidant properties than high molecular weight chitosan because of its improved solubility. This enhanced solubility facilitates the interaction of active functional groups with free radicals. In addition, the intramolecular hydrogen bonding of chitosan also affects its antioxidant performance. High molecular mass chitosan exhibits stronger intramolecular hydrogen bonding, leading to reduced activity of amino groups and hydroxyl groups in interacting with the external environment. In contrast, low molecular mass chitosan has a more relaxed structure, weaker intramolecular hydrogen bonding, and higher activity of amino groups and hydroxyl groups. This enhances its ability to react with free radicals, thereby enabling scavenging effects. Deacetylation, which refers to the presence of free amino groups in a sample, is also considered a crucial indicator of the antioxidant properties of chitosan.

### 3.4 Moisturizing

Chitosan has excellent moisturizing properties (Chaiwong et al., 2020), which can help retain the moisture of food products and prevent dryness and quality degradation caused by water evaporation. Due to the presence of many hydroxyl and amino groups in the chitosan molecule, these functional groups can form hydrogen bonds with water molecules, thereby enhancing the affinity of chitosan for water. Chitosan can also create a film on the surface of fruits to decrease water evaporation and enhance the moisturizing effect. The moisturizing effect of chitosan has mainly been demonstrated in cosmetics and skin care products (Chaiwong et al., 2022).

### 3.5 Biodegradability

Chitosan's degradability is one of its key properties, which means that it can be broken down by microorganisms into harmless substances in the natural environment. High utilization of thermoplastic polymers with low degradation rates as packaging materials results in the generation of large amounts of waste. Therefore, they should be replaced with natural polymers that can be degraded by microorganisms (Kaczmarek-Szczepańska, Sionkowska, Mazur, Świątczak, & Brzezinska, 2021). Chitosan consists of polysaccharides that can be broken down by various microorganisms, such as bacteria and fungi. Under suitable conditions (e.g., temperature, humidity, pH, etc.), microorganisms can utilize chitosan as a carbon and nitrogen source and decompose it into small molecules, such as carbon dioxide, water, and inorganic salts through processes such as enzymatic processes (Sina, Sarrafi, Tajbakhsh, & Fallah, 2023). Chitosan's degradability is related to its molecular weight and degree of deacetylation. In general, chitosan with a smaller molecular weight and higher deacetylation is more prone to degradation. Chitosan can dissolve in dilute acid to form a positively charged sticky electrolyte liquid, but it is unstable, and some long chains will be hydrolyzed. Its degradation products can provide carbon and nitrogen for the soil environment. Hydrolysis of chitosan leads to the production of monosaccharides with decreased viscosity and molecular weight. When the degree of deacetylation reaches 50%, chitosan transforms into a water-soluble substance that is fully biodegradable.

## 4 Application of chitosan in fruit preservation

Fruits are rich in nutrients. The external environment facilitates the growth of bacteria, making it easy for the product to rot during storage and transportation, leading to a decline in fruit quality or even corruption and deterioration (Zhong, Zhang, Tang, Adhikari, & Ma, 2023). The use of chitosan in fruit preservation involves two main methods: film wrapping and coating (Aswini, Azeez, & Duraikkannu, 2022). Film preservation involves the application of spraying, brushing, or dipping to create a thin film on the surface of fruits in order to maintain freshness. Film preservation involves utilizing chitosan's film-forming properties to create a protective film for wrapping fruits to facilitate preservation. Primarily, chitosan serves as the base material, with the addition of plasticizers and antimicrobial agents. Through intermolecular forces, a dense and stable film is formed during the composite process. The basic process of membrane production is as follows: dissolution of chitosan in a dilute acid solution → stirring → centrifugal filtration → defoaming → coating → drying → unveiling of the membrane. After the membrane is manufactured, various indicators of the membrane are tested, including viscosity, elongation at break, tensile strength, water permeability, and oxygen permeability.

### 4.1 Chitosan serves as a preservative alone

Among the available studies, chitosan emerges as a promising candidate for addressing the issues stemming from the extensive use of synthetic polymers in recent years. Chitosan, a polysaccharide with excellent antimicrobial and coating-forming properties, has attracted extensive research interest for its potential use as an edible coating for postharvest fruit preservation (Ruan, Kang, & Zeng, 2022). The preservation effect of chitosan varies with the same concentration. As the concentration of chitosan increases, its preservation effect will become increasingly evident. When the concentration reaches a certain level, the preservation effect will not increase significantly.

Lin et al. (2020) studied the effects of different concentrations of chitosan treatment on the storage and quality characteristics of harvested ‘Fuyan’ longan. Compared with the control samples, chitosan treatment in longan showed a lower fruit respiration rate, reduced peel cell membrane permeability, brown index, pulp decomposition index, fruit disease index, and weight loss. The results indicate that chitosan may be a simple and environmentally friendly post-harvest treatment method that can enhance the storage resistance of harvested longan fruit and extend its shelf life.

Xin, Jin, Chen, Lai, and Yang (2020) investigated the inhibitory effect of chitosan coating (2%) on softening and sodium carbonate soluble pectin (SSP) formation in sweet cherries during non-isothermal storage. The chitosan coating significantly prolonged softening time (6.4% higher than the control), maintained SSP content (6.6% higher than the control), and reduced SSP degradation by inhibiting the expression of the paPME1–5 gene, which regulates pectin Methyl esterase activity in sweet cherries under temperature changes. These results suggest that chitosan coating is feasible for preserving postharvest fruits under non-isothermal conditions. Triunfo et al. (2023) extracted chitosan from the black soldier fly was applied to the surface of ripe apricots, nectarines and yellow peaches and evaluated for physicochemical parameters. The results showed that the black soldier fly was a viable source of chitosan.

### 4.2 Chitosan and polysaccharide compound preservation

Chitosan itself has antibacterial functions, coupled with good biocompatibility, allowing it to be combined with various antibacterial agents and antioxidants. Additionally, through different modification processes of chitosan, its preservation performance can be further enhanced. Most of the studies have included polysaccharides as complexes. The combined use of chitosan with polysaccharides can enhance its stability, persistence, antimicrobial properties, and degradability. In addition, the interactions between different polysaccharides can also create synergistic effects to enhance the performance of materials.

Cellulose is the oldest and most abundant natural polymer on Earth. It is inexhaustible and the most precious natural renewable resource for human beings. Cellulose chemistry and industry began >160 years ago and were the primary focus of research during the emergence and advancement of polymer chemistry. The research results on cellulose and its derivatives have significantly contributed to the establishment, development, and enrichment of the discipline of polymer physics and chemistry. In the field of fruit preservation. Zhou et al. utilized hydroxypropyl methylcellulose (HPMC) as the intermediate layer to enhance the interface interaction between the chitosan (CS) coating and citrus, aming to enhance the preservation effect of citrus (Zhou et al., 2024). The results showed that the HPMC-CS double coating performed well in inhibiting fruit respiration, reducing decay rate and maintaining nutrient content. Notably, the HPMC-1.5% CS coating not only reduced the decay rate of citrus fruit by 45% and 31% but also maintained a higher ascorbate content compared to the uncoated group (CK) and pure CS coating. Ruan et al. introduced water-soluble dialdehyde cellulose (DAC) as a cross-linking agent for chitosan to enhance its stability. DAC/CS as the coating agent for citrus storage (Ruan et al., 2022). Chitosan has enhanced stability through biopolymers and will be a promising candidate material to be used as a green edible coating for fruit preservation.

Soluble soybean polysaccharides consist of polysaccharides like arabinogalactan, arabinogalactan, and acidic galactoglucan. They are less viscous than other biopolysaccharides and are known for their dispersibility, stability, emulsification, and adhesion properties. Soybean polysaccharides are effective in antioxidant, antibacterial, antiviral, and immunomodulatory activities. Liu et al. studied the preservation effect of a soluble soybean polysaccharide (SSPS)-based coating and a carboxymethyl chitosan (CMCS) and lavender essential oil (LEO) composite coating on bananas (Liu, Liu, Shao, Zheng, & Tang, 2022). SSPS/CMCS/LEO coating had an excellent antibacterial effect on both Gram-positive bacteria and Gram-negative bacteria. The antioxidant results show that LEO can significantly enhance the antioxidant activity of SSPS-based coatings. This study demonstrates that SSPS-based emulsion coatings, when combined with CMCS and LEO, have significant potential to prolong the shelf life of perishable fruits like bananas at room temperature.

Starch is a polymer carbohydrate and polysaccharides composed of a single type of sugar unit. Starch is widely used, with a focus on modified starch. Denatured starch refers to the alteration of the molecular structure and physical and chemical properties of native starch through physical, chemical, or enzymatic methods. This process aims to create new properties and applications for starch or its derivatives. Li et al. used starch and chitosan as membrane substrates, mulberry anthocyanins as indicators, and the delay method to prepare a new type of edible pH indicator membrane. The results of the performance test showed that by adding no mulberry anthocyanin composite membrane, they found that the formation of hydrogen bonds between mulberry anthocyanins and starch greatly improved the tensile strength of the composite membrane (Li, Sun, Gao, Yang, & Man, 2023). The addition of mulberry anthocyanins significantly improved the film thickness, opacity, tensile strength, and water vapor transmittance. However, it led to a significant reduction in the elongation of break, while the clearance of DPPH radical reached a maximum value of 85.24%. Other polysaccharides and chitosan compound preservation research is also very reference.

Zhao et al. prepared a composite plastic wrap by using chitosan and mushroom foot polysaccharide as the substrate. They characterized the structure and physicochemical properties of the composite plastic wrap using Fourier infrared spectroscopy, X-ray diffraction, and scanning electron microscopy (Zhao et al., 2023). With blueberries as the preservation object, the preservation effect of the composite film group was significantly higher compared to the control groups, which included the single chitosan film group and the uncovered group. The composite film group exhibited better antibacterial and antioxidant properties, effectively delaying fruit decay and extending the shelf life. Zeng et al. prepared lycopene microcapsules (LMs) using chitosan (CS) and carboxymethyl CS (CMCS) as wall materials. LM/SA/KGM composite films were prepared using sodium alginate (SA) and konjac glucomannan (KGM) as substrates (Zeng et al., 2022). The results showed that the composite film effectively extended the shelf life of sweet cherries, and effectively delayed the decrease in decay rate, pH, and soluble solids content.

### 4.3 Chitosan and protein compound preservation

The composite membrane with proteins as the substrate is formed through hydrogen bonds, disulfide bonds, hydrophobic interactions, etc. The composite membrane possesses specific mechanical and barrier properties. The -NH2 group of chitosan is positively charged and can interact electrostatically with the deprotonated carboxyl groups of proteins, thereby enhancing the antimicrobial storage stability of the composite membrane. Protein film-forming substrates mainly include plant isolates such as soy protein, gelatin, collagen, and animal isolates.

Soybean isolate protein is a high-quality soy protein food additive derived from low-temperature soluble soybean meal. It has a protein content exceeding 90%, contains nearly 20 types of amino acids, including essential amino acids, and offers high nutritional value. Additionally, it possesses excellent characteristics such as good low oxygen permeability, water resistance, and film-forming abilities. This protein can be utilized in fruit preservation to uphold fruit firmness and minimize the depletion of vitamins and other nutrients (Alves, Gonçalves, & Rocha, 2017). Chen, Ma, Li, and Tang (2023) used chitosan and soybean protein isolate (SPI) as the basic materials to create a safe, renewable, and degradable composite antibiofilm packaging material. At SPI addition of 0.66%, glycerol addition of 0.51%, and a drying temperature of 55 °C, the addition of ε-polylysine at 0.30% and 0.20% Nisin resulted in a significant antibacterial effect on the membrane. The preparation process of the renewable and degradable new packaging materials proposed in this study is relatively simple and offers better performance. This provides a new concept for developing environmentally friendly packaging materials in the future. Qi et al. explored the impact of varying concentrations of soybean protein on the structure and performance of chitosan-nanofiber edible membranes. They conducted research using Fourier infrared spectroscopy, X-ray diffraction, differential scanning calorimetry, and scanning electron microscopy to analyze the microstructure of the edible membranes, as well as to investigate the electrostatic effects and their influence on performance (Qi et al., 2023). The study concluded that adding 1% of SPI. The test results provide a theoretical reference for the preparation and application of proteoglycan edible membranes.

Whey protein is a valuable protein extracted from milk using advanced technology. It is promoted as the “king of protein” due to its advantages, such as high purity, high absorption rate, and the most balanced amino acid composition. Whey protein is considered a high-quality complete protein, as it is derived from animal sources. It contains 8 essential amino acids necessary for the human body, and the ratio is close to the proportion required by the human body. It is an essential substance indispensable for human growth, development, anti-aging, and other life activities (Pang et al., 2023). It was indicated that the optimal condition for forming the membrane was a chitosan concentration of 3%, a reaction temperature of 80 °C, and a pH of 5. Additionally, when comparing the appearance of uncoated film with that of film coated with whey protein monolayer and whey protein-chitosan bilayer on tomatoes, the bilayer film demonstrated a superior preservation effect.

Zein contains rich in sulfur amino acids, which are molecules with strong hydrophobic bonds and disulfide bonds. It exhibits good barrier properties, hydrophobicity, and membranogenicity. However, a single zein-coated membrane surface has higher hydrophobicity, but lacks high mechanical strength, making it brittle. Additionally, zein does not possess antibacterial properties, which limits its application in the field of coatings. By interacting with chitosan, the mechanical properties of the zein membrane can be enhanced, along with improved hydrophobicity, antibacterial ability, and maintenance of the appearance quality of the coated fruit. Zhang et al. (2019) prepared a series of chitosan/corn-soluble protein hybrid membranes with different ratios. It was found that the barrier effect of the blended membranes on water vapor, oxygen, and carbon dioxide was improved compared to that of single CS membranes. Hydrogen bonding between zeinolysin and chitosan (CS) molecules was observed, indicating good compatibility between the two. The addition of zeinolysin enhanced the thermal stability of the films.

### 4.4 Chitosan and essential oil compound preservation

Combining chitosan with essential oils, in addition to its recombination with polysaccharides, is also a good option. Plant essential oil has many biological activities and is a natural antibacterial agent that can replace chemical preservatives. This has garnered significant attention in the field of food preservation. Plant essential oils possess spectral bacteriostatic properties, and various essential oils, along with their active compounds, target different sites and operate through different modes of action. Different essential oils and their active substances have various sites and modes of action. For example, carvacrol in oregano essential oil can interrupt and penetrate the bacterial cell wall, causing cell membrane disruption and denaturation of cellular proteins (Oussalah, Caillet, Saucier, & Lacroix, 2007). Clove essential oil can inhibit the activity of specific enzymes required by bacteria, such as ATPase in bacteria or enzymes involved in cell wall synthesis in molds. It can also disrupt the synthesis of nucleic acids within the cell (Nisar et al., 2018). Some enzymes in bacteria or mycobacteria are involved in cell wall synthesis and can disrupt intracellular nucleic acid synthesis. Cinnamaldehyde, found in cinnamon essential oil, can penetrate the cell wall and inhibit the synthesis of dextran and chitosan in the cell wall. This leads to damage to the cell wall and eventual cell death due to the loss of barrier protection (Yin et al., 2020). The application of natural plant essential oils as antimicrobial agents effectively controls the growth of spoilage and pathogenic microorganisms. Therefore, chitosan edible coatings and films containing essential oils expand the widespread use of antimicrobial packaging in food products (Zhang et al., 2021).

Martínez et al. (2018) utilized chitosan in conjunction with essential oils to create an edible coating. This coating was used to maintain the freshness of strawberries. Various concentrations of thyme essential oil were applied to the chitosan coating, resulting in diverse effects on the physical and microbiological characteristics of the strawberries. All products exhibited higher acceptability and quality compared to the control product, with the treatment group that included essential oils showing the most effectiveness. Wang, Zhang, Dong, and Wang (2022) investigated the effect of chitosan (1.0%, *w*/*v*) and tephrosia essential oil (TEO) on loquat fruit quality at concentrations of 0.5% and 1.0% (*v*/v) treatments. The results showed that the treatment with 1.0% chitosan +1.0% TEO delayed the adverse postharvest changes and maintained the quality of loquat, demonstrating the feasibility of loquat preservation. Long et al. (2022) prepared six types of composite films with chitosan, fennel seed essential oil (FEO), and starch octenyl sodium succinate (SSOS) to maintain the freshness of apples. Its physicochemical, antifungal, and antiseptic properties were investigated. It was found that the best ratio for SSOS/glycerol/FEO/chitosan addition was 1:0.2:1:0.5. Phuong, Van, Nkede, Tanaka, and Tanaka (2024) studied the effects of two different edible coatings: chitosan and chitosan-loaded lemongrass essential oil on the quality parameters of strawberries. CH and CH + LMO coatings can effectively extend the shelf life of strawberries and control their quality characteristics. Alvarez-Perez et al. (2023) preserved Anju pears using a chitosan bag infused with cinnamon leaf essential oil (CLEO) and assessed the antifungal properties of Anju pears during refrigeration and room temperature storage. CLEO affects the content of polyphenol oxidase and total phenols. Chitosan bags containing CLEO can better preserve pear fruit, thereby reducing weight loss, maintaining maximum hardness, and preserving good color. Yuan et al. (2023) combined cinnamon essential oil (CEO), titanium dioxide, and chitosan (CS) to prepare a safe and renewable nanocomposite (CS-TC) for fruit preservation. Strawberry coated with CS-TC had higher hardness, lower weight loss, and mildew rate, and a shelf life 4 days longer at 20 °C compared to the control.

### 4.5 Chitosan-nanomaterials composite preservation

Through research on the application of chitosan in coating fruits and vegetables to preserve freshness, it was discovered that chitosan, as a novel type of coating agent, exhibits potent antimicrobial activity. The low strength of chitosan film in wet state affects its mechanical properties. The insufficient strength and toughness of the film lead to easy breakage after use, exposing the surface layer of fruits and vegetables (Yang et al., 2023). This issue hinders its wide range of applications and is attributed to chitosan's inherent structure and properties. Nano TiO_2_ is one of the most active inorganic nanomaterials. It possesses characteristics such as non-toxicity, antibacterial properties, decomposition of bacteria, ultraviolet protection, superhydrophilicity, and superhydrophobicity. It is extensively utilized in cosmetics, antibacterial fibers, and other fields. To address the issue of chitosan as a film and enhance its applicability in fruit preservation. Liu et al. (2021) characterized the litchi peel extract (LPE) using LC-MS/MS. When chitosan (CS), nano-TiO2, and low-density polyethylene (LPE) were utilized to create the active packaging membrane coating, nano-TiO2 influenced the physical and mechanical structural properties of the film. Polyphenol oxidase activity, electrolyte leakage, and malondialdehyde accumulation were measured after treatment. CS + TiO_2_ + LPE as a food fruit preservative. Xing et al. (2020) prepared a chitosan/titanium dioxide nanocomposite with sodium lauric acid-modified nano-TiO2 as the main film-forming material (nano-TiO2) and studied the effect of the chitosan/nano-TiO2 complex on the physiology of mangoes after harvest. The results showed that the film mulching enhanced the firmness of the mango, which was 5.9 kg/cm^2^ higher than the uncoated fruit, and played an important role in maintaining the fruit quality. Qiao, Xiao, Ding, and Rok (2019) studied the cooling effect of chitosan/nano titanium dioxide coated with an antibacterial agent on ready-to-eat cantaloupe fruit. Ascorbic acid and juice leakage from (CH/TiO_2_) treatment were significantly reduced by the (CH/TiO_2_) treatment compared to uncoated samples. Phenol oxidase (PPO) activity was much lower than that of the control sample fruit. Sami, Soltane, and Helal (2021) studied the characteristics of a new chitosan/silica nanoparticles/streptococcus lactate film, in which streptococcus ate was added as an antibacterial technology for blueberries during storage. Chitosan/silica nanoparticles (N(CH-SN-N)) present a stable suspended surface load that reduces microbial contamination in blueberry coatings. Eldib, Khojah, Elhakem, Benajiba, and Helal (2020) applied a chitosan coating with silica nanoparticles and lactobacillus peptide to fresh blueberry samples to conduct in vitro studies on fruit quality, focusing on oxidative physicochemical parameters, and in vivo analysis of mold/yeast microflora. The results show that the nanomaterial thin film coating can effectively extend the shelf life of blueberries.

### 4.6 Application of modified chitosan in fruit preservation

Due to its biodegradability, chitosan is an intriguing alternative material for packaging development. Although chitosan exhibits antimicrobial activity against a variety of microorganisms, its solubility is restricted to acidic solutions with a pH <6.5, thereby constraining its potential application as an antimicrobial agent. Water solubility is the decisive factor in determining whether chitosan can exhibit antibacterial resistance. Therefore, modifying chitosan to enhance water solubility and antibacterial resistance has become a prominent topic in recent years. The modification process of chitosan includes acid degradation, enzymatic degradation, oxidative degradation, physical degradation, and other methods (Wang et al., 2023). The study confirmed that the antibacterial performance and stability of the modified chitosan composite membrane were significantly enhanced.

To enhance the surface hydrophobicity of chitosan molecules, some researchers have increased the solubility of chitosan in water by introducing carboxymethyl groups (Pan et al., 2022). The reaction of chitosan with chloroacetic acid under alkaline conditions produces carboxymethyl chitosan (Taubner, Marounek, & Synytsya, 2020), which is the most commonly used chitosan derivative today and exhibits higher antibacterial activity than chitosan. The solubility of chitosan increases with carboxyalkylation, and the presence of the carboxyl group lowers the pH, which facilitates aminoprotonation. Carboxymethylation of chitosan occurs not only on the -OH group but also involves substitution on the -NH2 group. The reaction conditions must be strictly controlled to obtain carboxymethyl chitosan with a consistent structure and to impact its antimicrobial activity (Skorik, Pestov, Kodess, & Yatluk, 2012). When reacting under alkaline conditions, the activity of the carboxymethylation reaction is as follows: the activity of the primary hydroxyl group is greater than the activity of the secondary hydroxyl group, which is greater than the activity of the amino group. Sun, Wei, and Xue (2023) prepared a composite membrane with carboxymethyl chitosan (CMCS), pectin, blueberry anthocyanins (ACNs), and clove oil. It was used for strawberry preservation, and the strawberries maintained their good morphological structure after seven days of storage. Yang et al. (2023) synthesized amphiphilic chitosan and carboxymethyl-modified gellan gum to develop an active edible preservation material for mangoes. The composite film demonstrated effective preservation of mango fruits during 20 days of storage. Therefore, the multifunctional composite film can be used as a promising environmental protection packaging material for fruit preservation. The polymeric films of carboxymethyl chitosan (CMCs) and carboxymethyl starch (CMS) containing date kernel (DK) extract were prepared, intending to apply it as a material that can kill microbes that lead to spoilage of preserved fruits. Results revealed that the CMCs/CMS/DK-3 had superior antimicrobial possibilities against all selected microbes without any hazardous effect while applied to the microbially contaminated solution (Zidan et al., 2023).

Quaternization is another modification methods used to improve the water solubility and antimicrobial properties of chitosan. There are generally two ways to achieve chitosan quaternization: (1) direct modification of the amino groups on the chitosan backbone to quaternary ammonium salts; (2) introduction of quaternary ammonium groups outside the chitosan backbone (Jin et al., 2022). This quaternization method allows for the introduction of hydrocarbon groups with varying carbon chain lengths into the molecular structure of chitosan, leading to the formation of chitosan quaternary ammonium derivatives with different carbon chain lengths. The water solubility of the quaternized chitosan increases, leading to an increase in antimicrobial activity. Carboxymethyl chitosan quaternary ammonium salt (HACC) exhibited the highest antibacterial activity among these derivatives. Its antibacterial effectiveness increased with the rise in positive charge. This indicates the crucial role of positive charge in chitosan quaternary ammonium salt in bacterial inhibition. Pan et al. synthesized hydroxypropyltrimethyl ammonium chloride chitosan (HACC) (Pan et al., 2022). Subsequently, they prepared a multifunctional food packaging composite film with good thermal stability and antimicrobial function by modifying the quaternized chitosan quaternary ammonium salt (HACC) with HA-CS-NP and polyvinyl alcohol (PVA). The freshness of mangoes and papayas was preserved, the final hardness value was improved, and the coated film has a broad application prospect in fruit packaging. Jiang et al. (2024) developed a hexanal-loaded ZIF-8/CS nanocomposite film (HZCF) with a “nano-barrier” structure by growing ZIF-8 co-crystals in situ on quaternized chitosan (CS) and encapsulating hexanal into ZIF-8 via microporous adsorption. Contributed to the multifunctional features, HZCF prolonged the shelf life of banana and mango for at least 16 days, which is 8 days longer than that of control fruit. Fu et al. (2021) used poly (lactic acid) (PLA) and poly (vinyl alcohol) (PVA)-quaternized chitosan as the matrix for the outer and inner layers, respectively, to prepare bilayer films by solution casting; and CuO @ ZIF 8 nanoparticles were introduced into the PVA - quaternized chitosan layer. The freshness preservation test of cherry tomato showed that the composite film retarded the growth of harmful microorganisms on the fruit surface.

Glycosyl modification enhances the water solubility of chitosan and confers more new properties (e.g., targeting) to chitosan derivatives (Chen et al., 2022). Therefore, glycosyl modification is an effective method to obtain chitosan derivatives with multifunctionality and good water solubility (Panahi et al., 2023). D-Glucosamine and *N*-acetyl-D-glucosamine were selectively grafted onto C6 of chitosan. The antimicrobial properties of the products were determined, and the results showed that glycosylated chitosan derivatives exhibited stronger antimicrobial properties against *Bacillus subtilis* and *Staphylococcus aureus* than chitosan. Moreover, glucosamine-modified chitosan demonstrated higher antibacterial activity than mannose-modified chitosan, highlighting the significance of the free amino group.

## 5 Factors affecting the preservation effect of chitosan

### 5.1 Deacetylation degree

Degree of deacetylation (DD) is the ratio of the number of glucosamine units with the acetyl group removed to the total number of glucosamine units (Qin et al., 2023), and it is one of the fundamental structural parameters for analyzing chitin/chitosan. Deacetylation degree has a significant impact on the solubility properties, viscosity, ion exchange capacity, and flocculation properties of chitosan (Yu, Zhang, Guo, & Qian, 2023). Usually, chitosan with >55% of the *N*-acetyl group removed can be soluble in 1% acetic acid or hydrochloric acid and is called chitosan. However, chitosan with more than a 70% deacetylation degree can only be used as a valuable industrial product. Chitosan with deacetylation degrees of 55%–70%, 70%–85%, 85%–95%, and 95%–100% are referred to as low-deacetylated chitosan, medium-deacetylated chitosan, high-deacetylated chitosan, and ultra-high-deacetylated chitosan, respectively. It is extremely challenging to prepare chitosan with a deacetylation degree of 100% (Wang, Wang, Xie, & You, 2022). During deacetylation, the acetyl group (-C2H3) is replaced by the amino group (−NH2) on the polymer chain to form copolymers of *N*-acetyl-glucosamine and D-glucosamine. The higher the degree of deacetylation in the chitosan molecule, the more amino groups it contains. This results in a higher degree of molecular chain, increased solubility, lower molecular weight, and enhanced biological activity. Therefore, the degree of deacetylation is one of the key factors influencing the physical, chemical properties, and biological activity of chitosan. Yang et al. (2021) investigated the effect of chitosan deacetylation degree on the gel properties and structural characterization of microcrystalline cellulose-polysaccharide-chitosan (MCP-CH) hydrogels. With the increase in DD, the viscoelasticity, water-holding capacity, and thermal stability of the hydrogels improved. With the formation of MCP-CH hydrogels, new crystallization peaks appeared. The honeycomb microstructure of MCP-CH hydrogels improved with increasing DD. Nowadays, it has been investigated that the degree of deacetylation of chitosan can be accurately determined by liquid chromatography-electrospray ionization-mass spectrometry (LC-ESI-MS/MS) (Xue et al., 2022).

### 5.2 pH

Many studies have shown that the antimicrobial activity of chitosan is enhanced with increasing solution acidity, which may be attributed to the polycationic nature of chitosan. In general, the solubility and antimicrobial properties of chitosan may be enhanced under acidic conditions, making it more effective in fulfilling its preservation role in environments with lower pH levels. A low pH may specifically facilitate the protonation of the amino groups of chitosan, increasing its positive charge density. This, in turn, enhances its interaction with microbial cell surfaces and inhibits microbial growth. In addition, an acidic environment may also help chitosan form more stable complexes and enhance its preservation effect. It was found that the bacterial inhibition effect of chitosan began to weaken at pH levels higher than 6.5, and the optimal bacterial inhibition effect was achieved at pH 5.0–5.5.

### 5.3 Molecular mass and mass concentration

Molecular mass and mass concentration are important factors that affect the preservation effect of chitosan. It was found that the freshness preservation effect of chitosan improves as the molecular weight increases (Li et al., 2023; Li, Sun, et al., 2023). However, once the molecular weight reaches a certain level, the enhancement of its effect becomes less noticeable. Jiang et al. electrostatically sprayed chitosan coatings with different number average molecular weights (MWs) of approximately 5, 19, and 61 kDa on strawberries (Jiang et al., 2020). The results showed that the 61 kDa CH coating was more effective in retarding the increase in pH and malondialdehyde (MDA) content, and better maintained the flavonoid content. Zhang, Cao, and Jiang (2022) analyzed the film-forming properties of chitosan with different molecular weights (30, 100, 200, and 300 kDa) and their adhesion properties to the surfaces of various post-harvest fruits. The chitosan films with higher molecular weight were observed to have better water resistance, water vapor barrier, and mechanical properties.

## 6 Summary and outlook

This paper summarizes the application of chitosan in the field of fruit preservation. The structure, preparation methods and physiological activity of chitosan are briefly summarized. The application of chitosan in the field of fruit preservation is summarized from six aspects: chitosan alone, combined with polysaccharides, combined with proteins, combined with essential oils, combined with nanomaterials and modified chitosan. Finally, the factors affecting the preservation effect of chitosan are briefly described.

In recent years, with the continuous improvement of people's living standards and the rapid development of the food industry, public concern for food safety is also increasing. Chemically synthesized preservatives are unable to meet people's needs. Chitosan, as a new type of food preservative, has a broad range of potential applications. Chitosan edible film is an ideal food packaging material and a new type of functional additives. It can effectively inhibit the growth of pathogenic bacteria and food-borne microorganisms, broadening its application field. The excellent characteristics of chitosan have been widely utilized. With the passage of time, its application will become more and more extensive. At present, it has made significant progress in research and application in the packaging and anticorrosion of food. It effectively slows down the deterioration and decay of food, reduces enzymatic browning, minimizes water loss, and preserves the color, taste, and texture of food. Consequently, it helps maintain the commercial value of food. In today's social development, people are increasingly demanding safe, healthy, and environmentally friendly lifestyles. Chitosan, with excellent antimicrobial properties, good biocompatibility, and natural alkalinity, is likely to be well-received by the public. Its superb adsorption and hygroscopicity make it suitable for various applications in textile materials, composites, and other fields. For instance, in the medical field, where fabrics need to exhibit good biocompatibility and antibacterial properties. Chitosan is a versatile fabric modifier known for its compatibility and antimicrobial properties. It is commonly used in health textiles for its health care benefits and antimicrobial properties. Additionally, chitosan is utilized as a preservative in agriculture and food industries. Moreover, it serves as an environmentally friendly option for wastewater adsorbents.

Chitosan, as a novel food preservative material, holds great potential for various applications, but its performance in all aspects still requires further enhancement. From the existing research results, the preparation method of this material remains challenging, and the current production cost is high. In future research, a variety of new preservation methods, such as ultrasonic pretreatment, enhanced freeze-drying, irradiation, and electrostatics, will be utilized to further enhance the preservation effect of the product.

## CRediT authorship contribution statement

**Jingjing Wang:** Writing – original draft, Methodology, Investigation, Formal analysis, Data curation. **Yuning Yuan:** Investigation, Validation, Writing – original draft. **Yu Liu:** Writing – original draft, Validation, Software, Investigation. **Xiang Li:** Investigation, Supervision, Writing – review & editing. **Shengjun Wu:** Writing – review & editing, Resources, Project administration, Funding acquisition, Conceptualization.

## Declaration of competing interest

None.

## Data Availability

The authors do not have permission to share data.
